# Supplementary material for: Investigation of the Storage and Stability as Well as the Dissolution Rate of Novel Ilaprazole/Xylitol Cocrystal
Source: Pharmaceutics. 2024 Jan 17;16(1):122. doi: 10.3390/pharmaceutics16010122 (PMC10818325; doi:10.3390/pharmaceutics16010122)
Supplement: Supplementary file 1 [file pharmaceutics-16-00122-s001.zip › pharmaceutics-2831426-supplementary.pdf]

Supporting Information

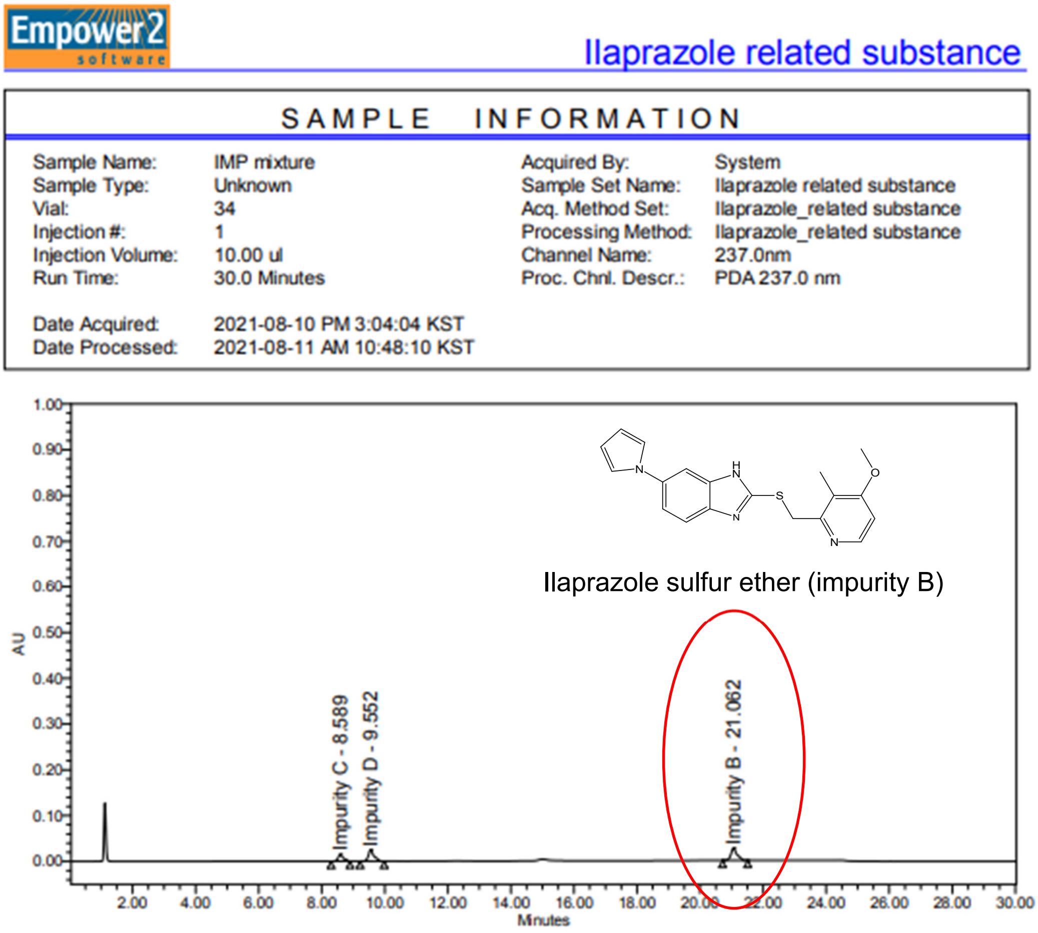

	Peak Name	RT	Area	% Area	Height
1	Impurity C	8.589	170673	20.44	15180
2	Impurity D	9.552	305479	36.59	25735
3	Ilaprazole	15.780			
4	Impurity B	21.062	358679	42.96	27079

Figure S1. Shows the chromatogram of Ilaprazole (Ila) impurity obtained through High-performance liquid chromatography (HPLC) analysis, depicting the retention time (RT). (RT: 21.062 min)

Empower 2 software

Ilaprazole related substance

SAMPLE INFORMATION

Sample Name:	STD_2	Acquired By:	System
Sample Type:	Unknown	Sample Set Name:	Ilaprazole related substance
Vial:	36	Acq. Method Set:	Ilaprazole_related substance
Injection #:	1	Processing Method:	Ilaprazole_related substance
Injection Volume:	10.00 ul	Channel Name:	237.0nm
Run Time:	30.0 Minutes	Proc. Chnl. Descr.:	PDA 237.0 nm
Date Acquired:	2021-08-10 PM 4:05:57 KST		
Date Processed:	2021-08-11 AM 10:48:27 KST		

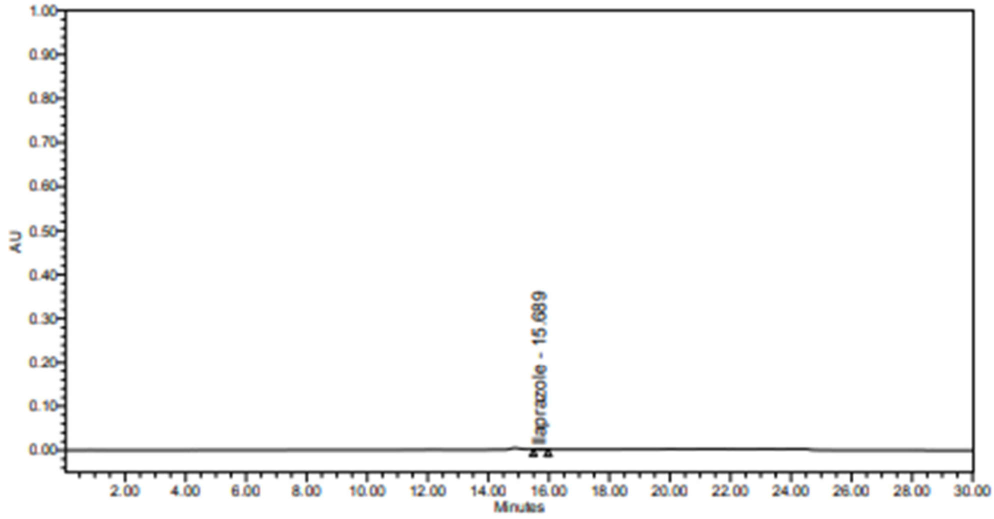

	Peak Name	RT	Area	% Area	Height
1	Impurity C	8.580			
2	Impurity D	9.550			
3	Ilaprazole	15.689	7422	100.00	683
4	Impurity B	21.600			

Figure S2. Shows the chromatogram of Ilaprazole (Ila) obtained through High-performance liquid chromatography (HPLC) analysis, depicting the retention time (RT). (RT: 15.689 min)

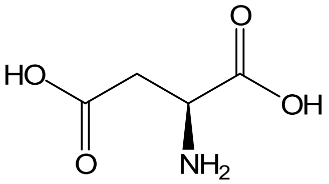

(a)

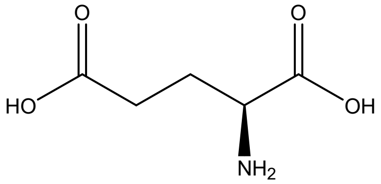

(b)

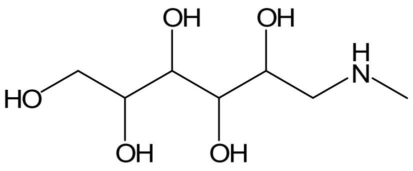

(c)

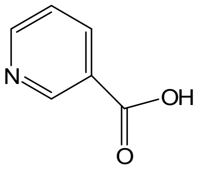

(d)

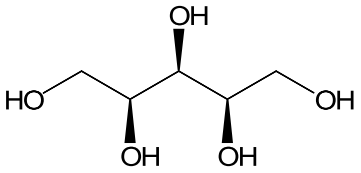

(e)

Figure S3. The molecular structures of the coformers presented in Table 1; (a) L-Aspartic acid (Asp); (b) L-Glutamic acid (Glu); (c) Meglumine (Meg); (d) Nicotinic acid (Nic); (e) Xylitol (Xyl)

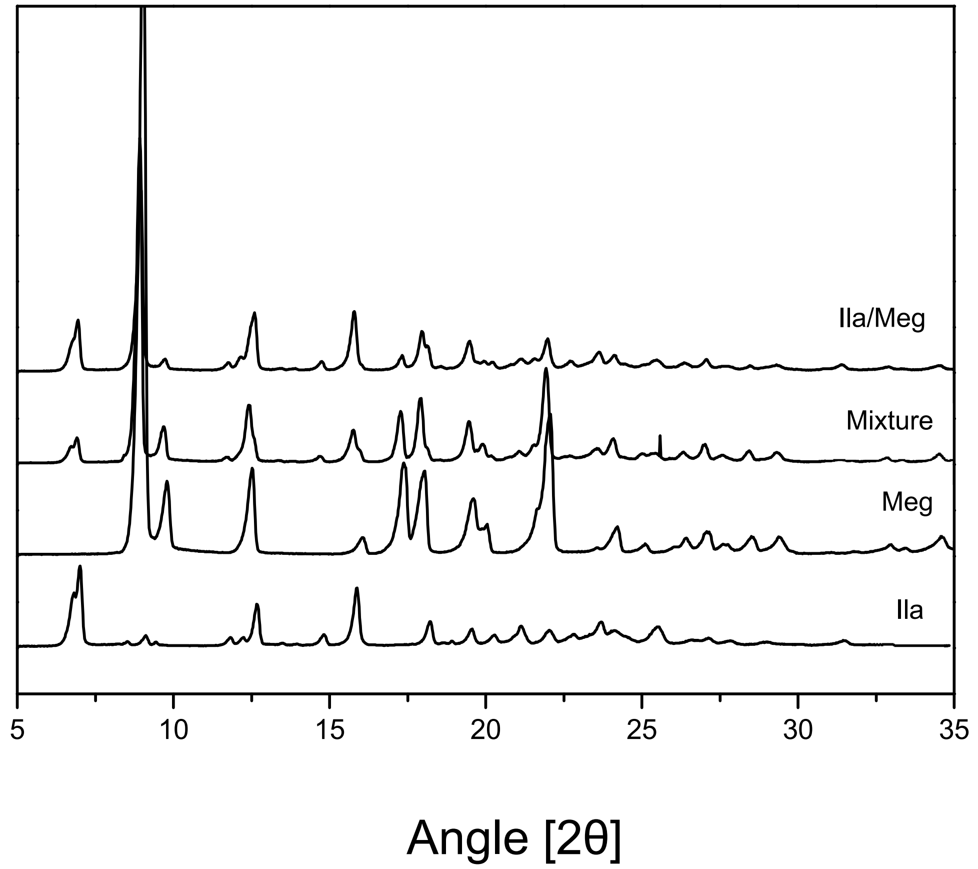

Figure S4. PXRD pattern of Ila, Meg, Ila/Meg physical mixture and Ila/Meg obtained by co-crystallization.

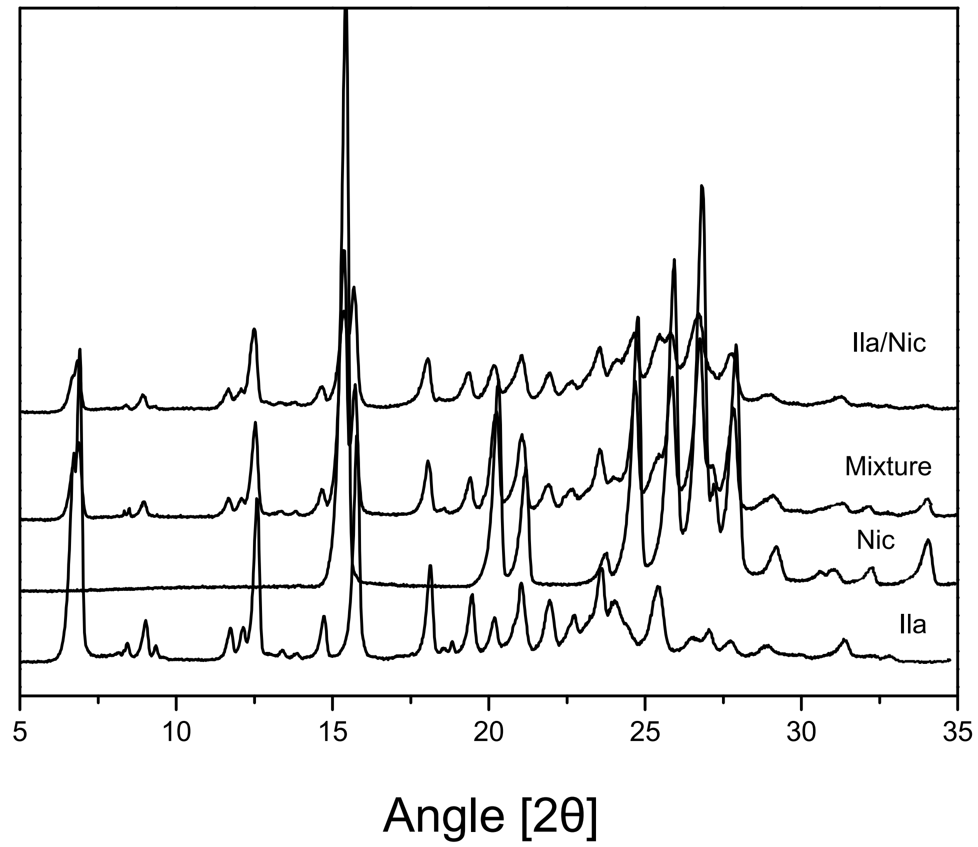

Figure S5. PXRD pattern of Ila, Nic, Ila/Nic physical mixture and Ila/Nic obtained by co-crystallization.

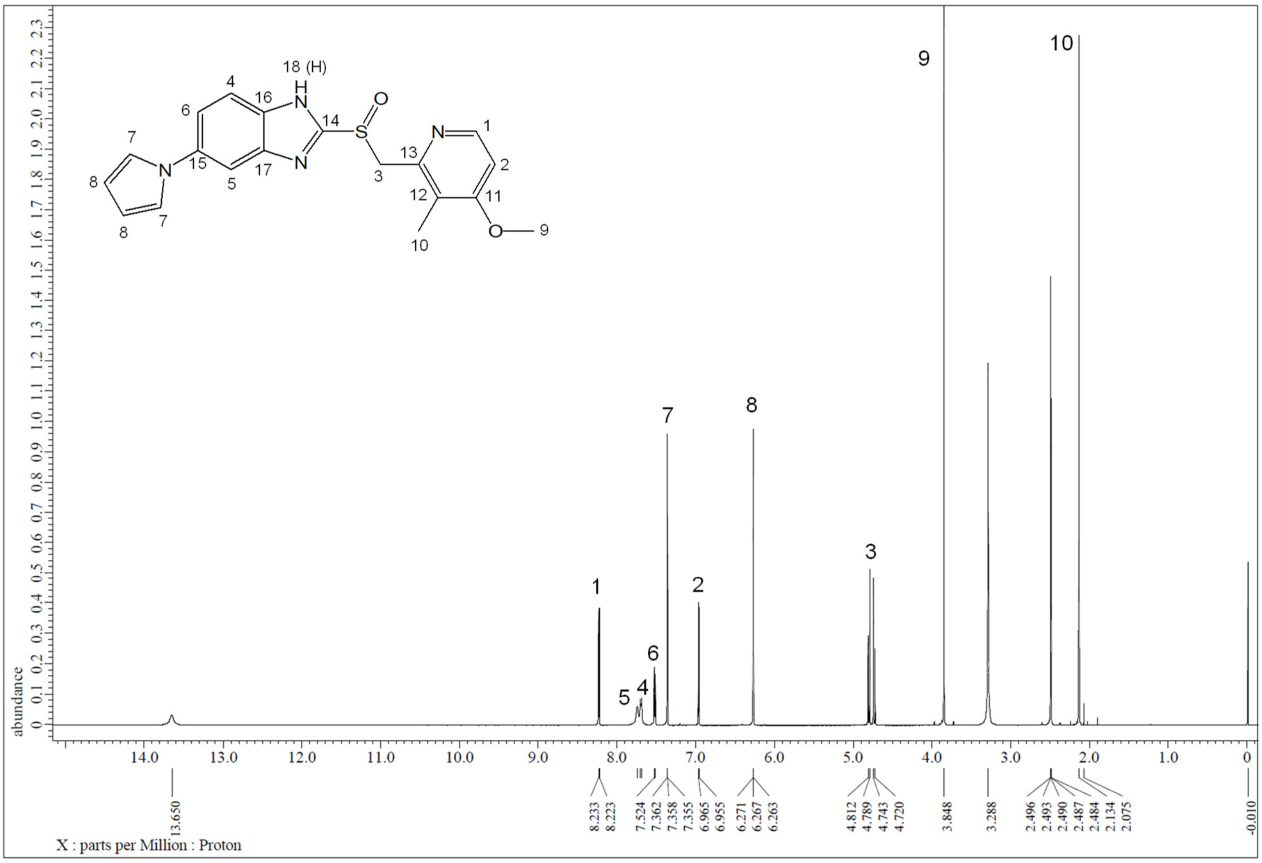

Figure S6. Solution-state ^{1}H-NMR of Ilaprazole (Ila) (solvent: DMSO-d^{6}) (Hydrogen numbering for each peak has been completed.)

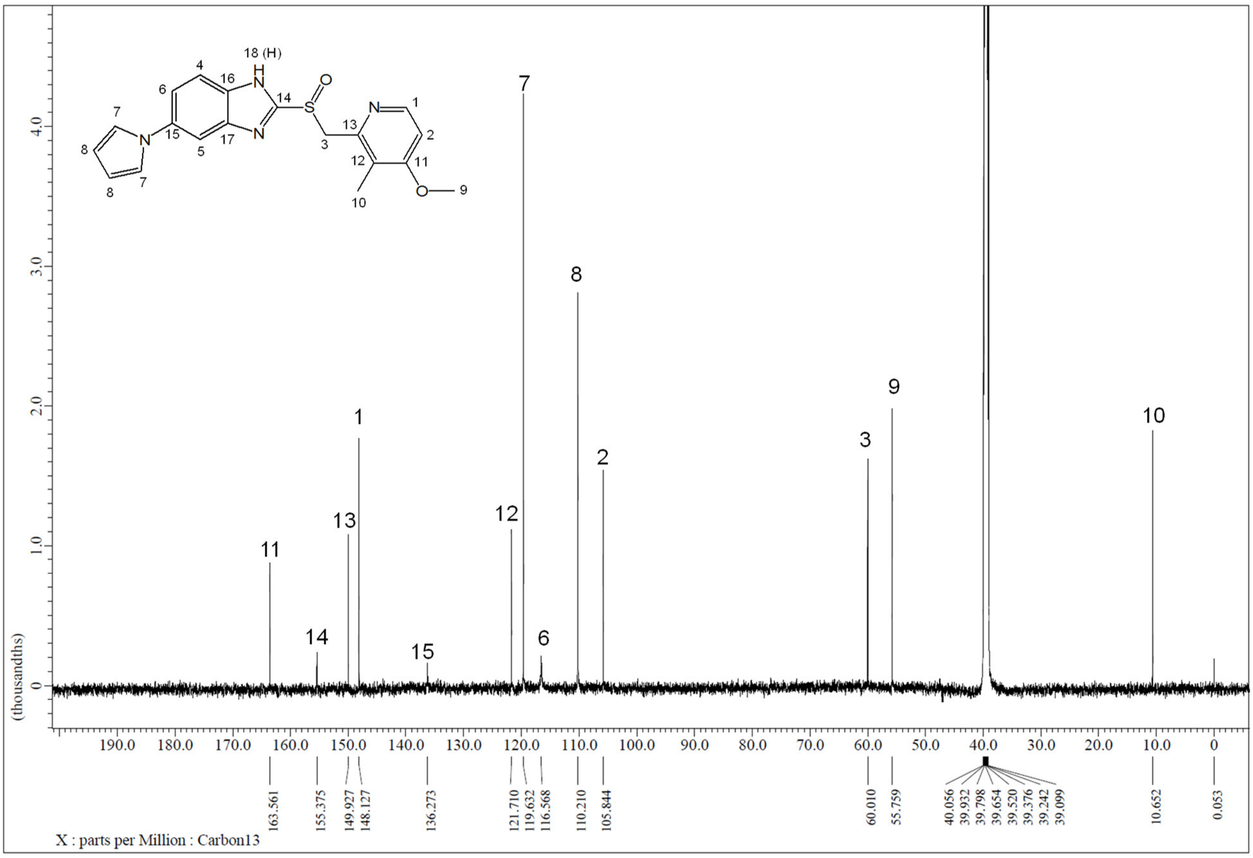

Figure S7. Solution-state ^{13}C-NMR of Ilaprazole (Ila) (solvent: DMSO-d^6) (Carbon numbering for each peak has been completed.)

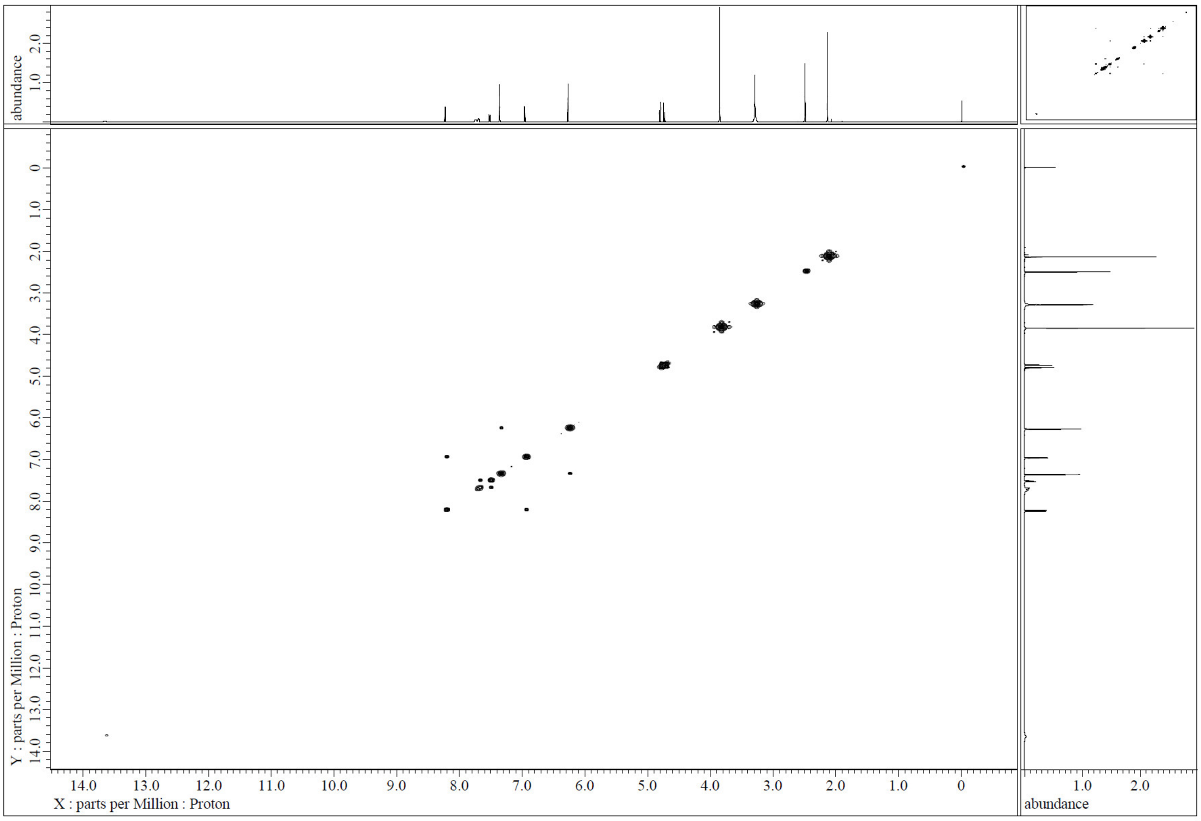

Figure S8. Solution-state 2D NMR (^1H-^1H COSY) of Ilaprazole (Ila)

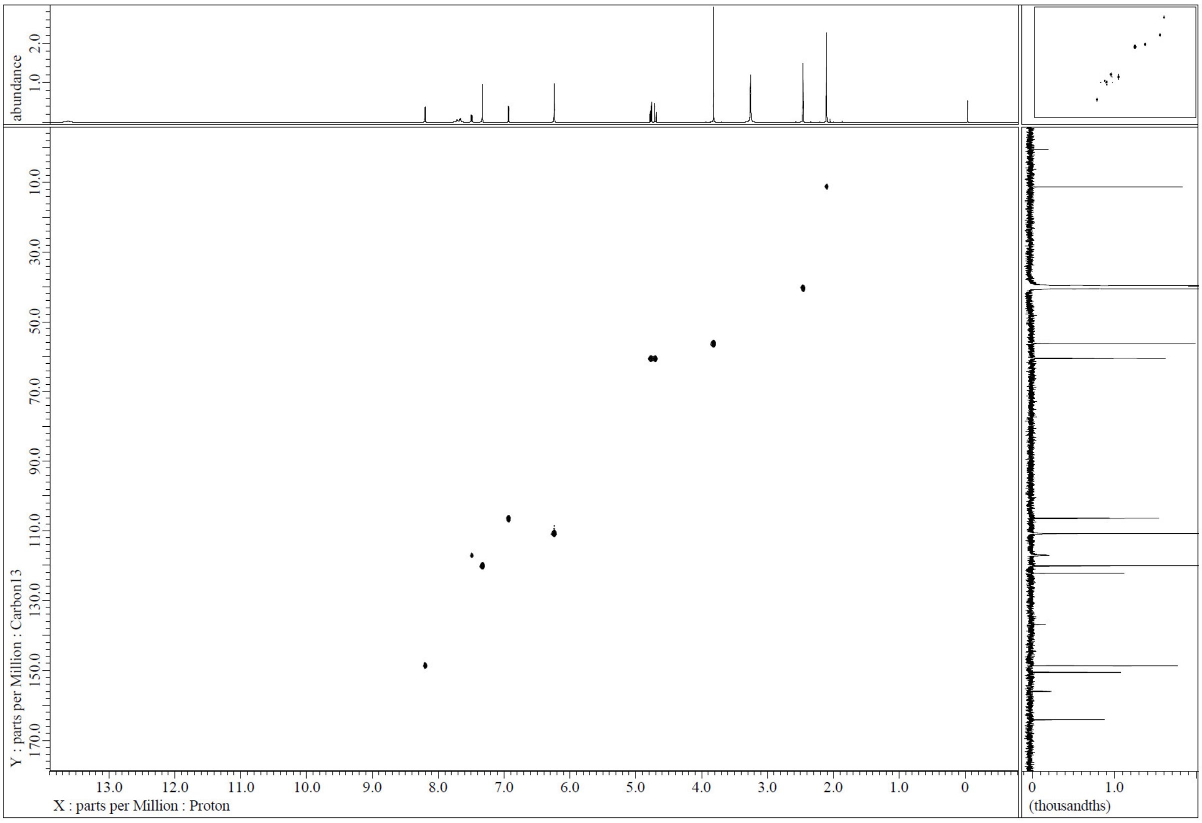

Figure S9. Solution-state 2D NMR (^1H-^{13}C HSQC) of Ilaprazole (Ila)

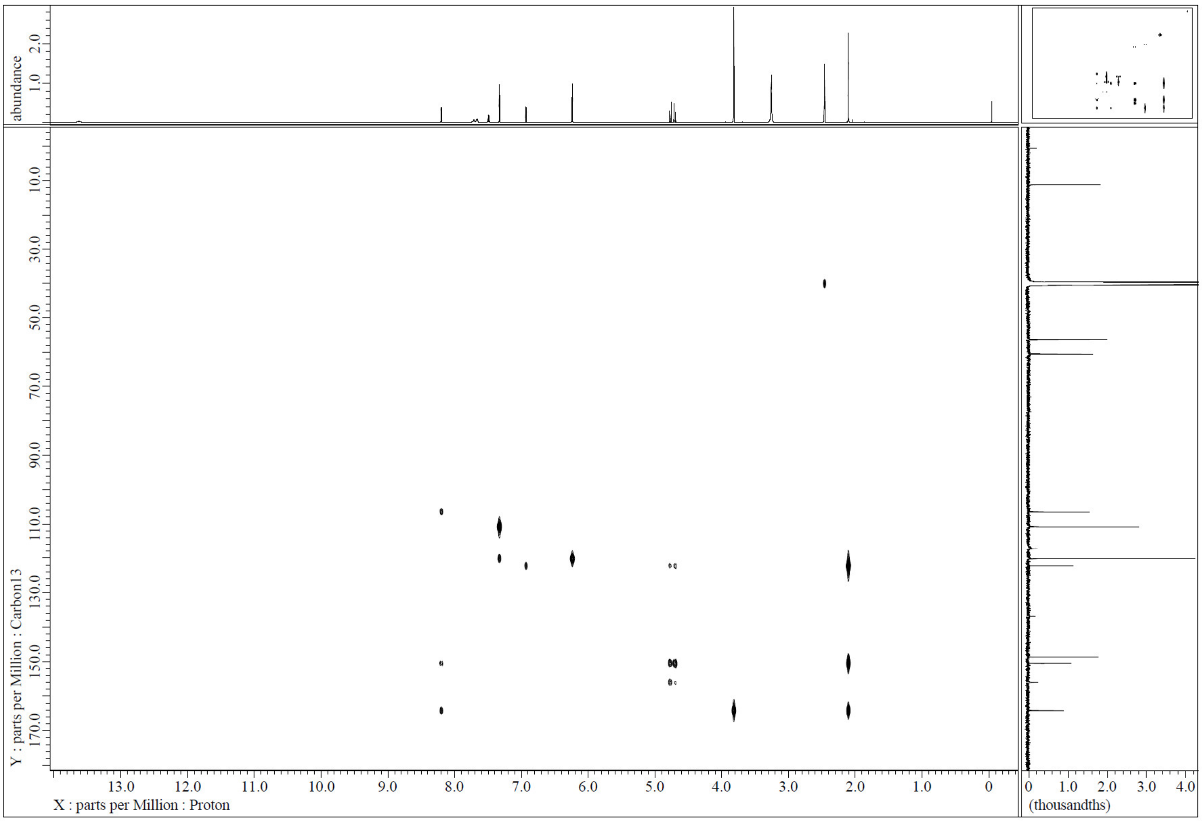

Figure S10. Solution-state 2D NMR (^1H-^{13}C HMBC) of Ilaprazole (Ila)

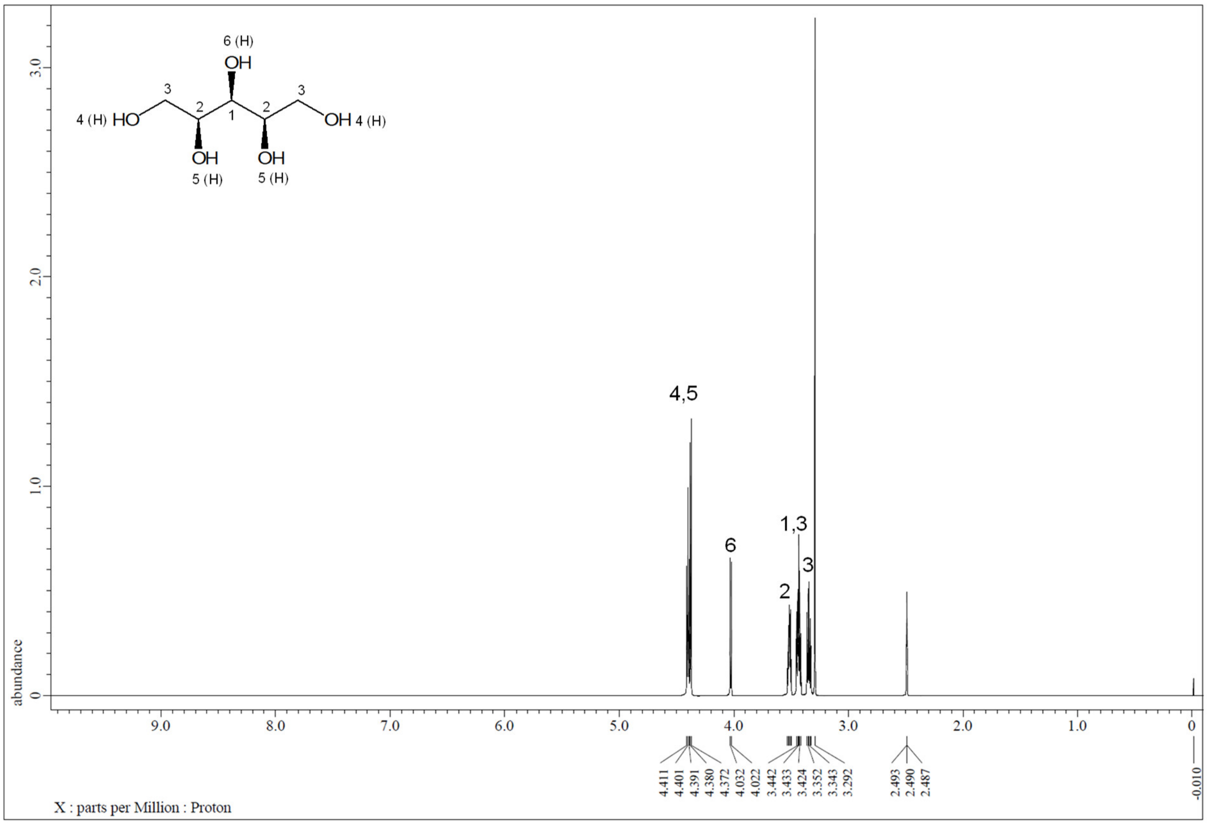

Figure S11. Solution-state ^{1}H-NMR of Xylitol (Xyl) (solvent: DMSO-d^6) (Hydrogen numbering for each peak has been completed.)

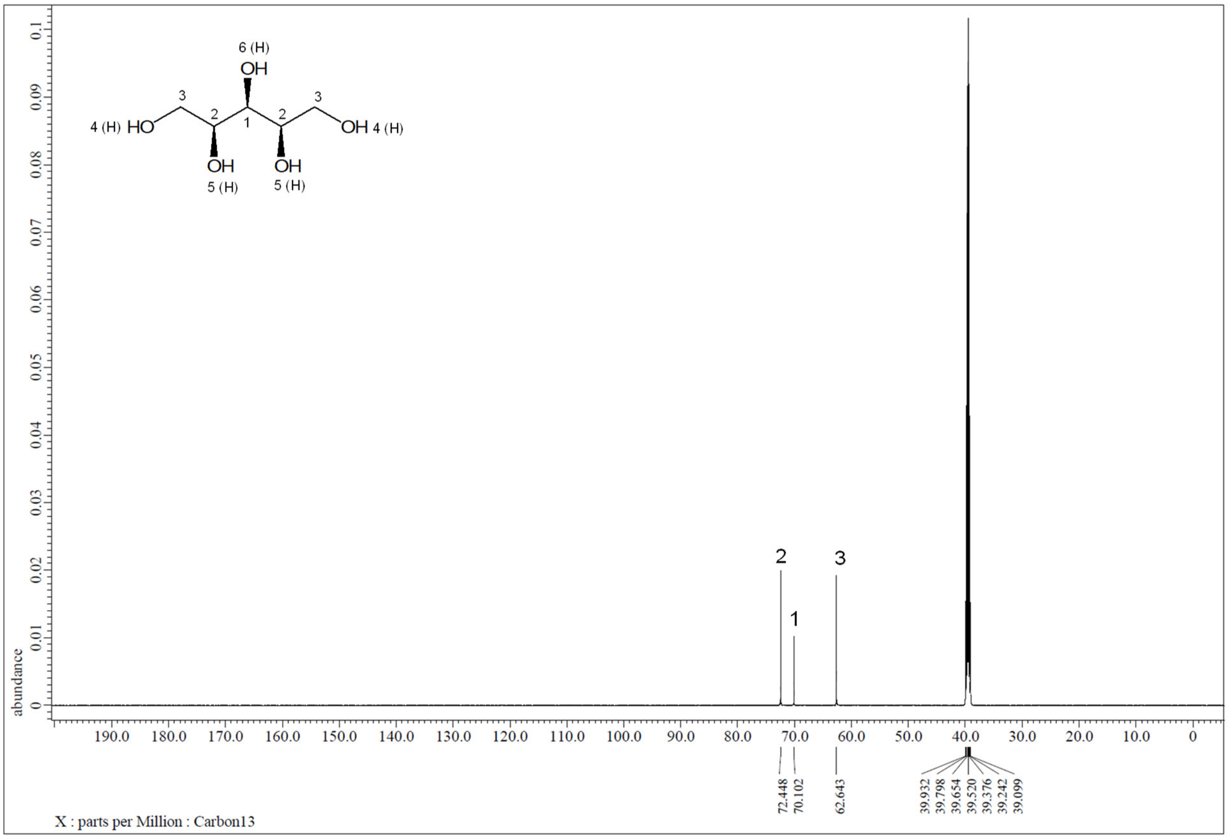

Figure S12. Solution-state ^{13}C-NMR of Xylitol (Xyl) (solvent: DMSO-d^{6}) (Hydrogen numbering for each peak has been completed.)

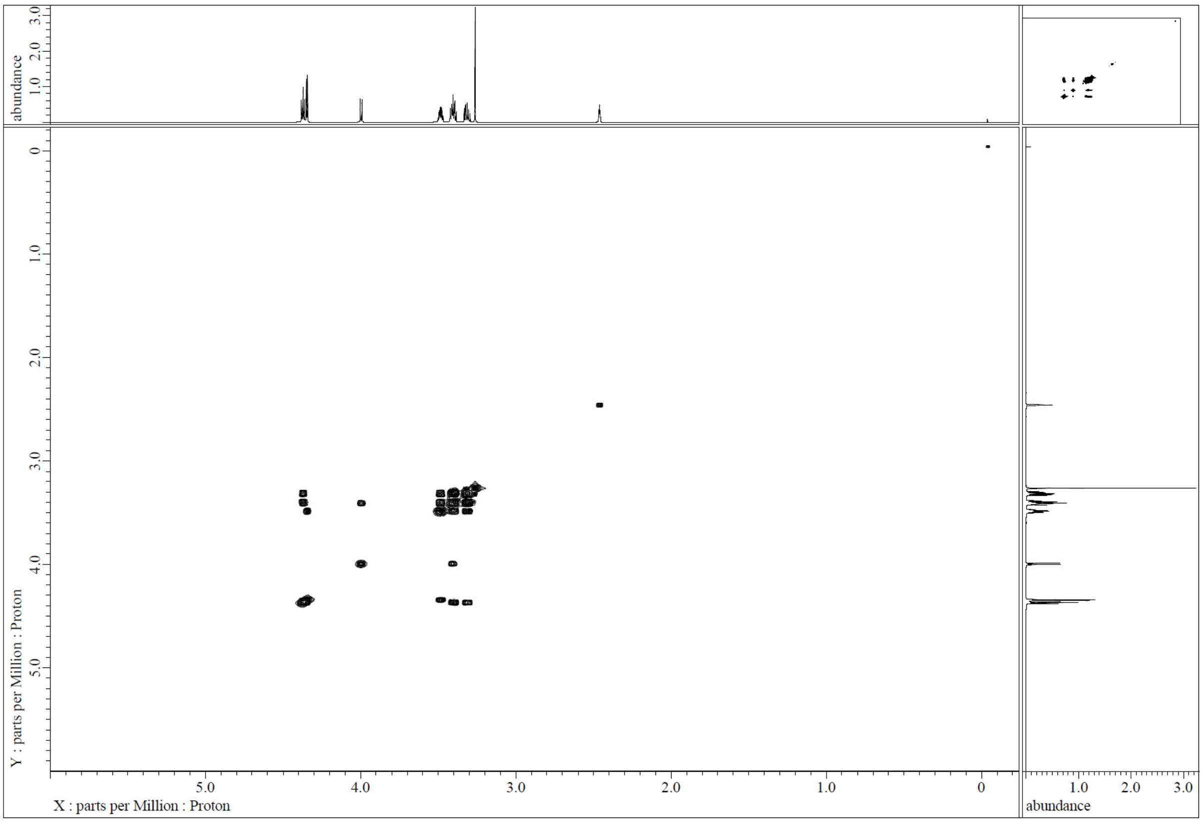

Figure S13. Solution-state 2D NMR (^1H-^1H COSY) of Xylitol (Xyl)

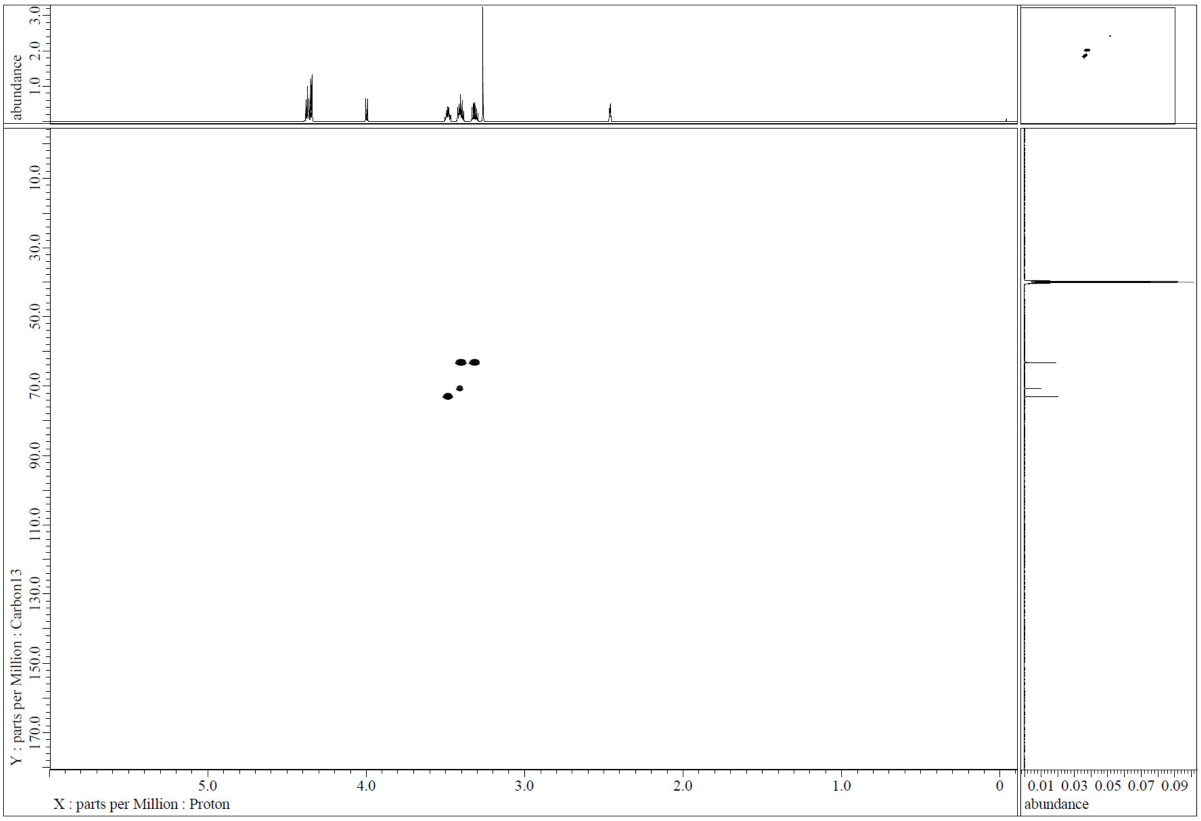

Figure S14. Solution-state 2D NMR (^{1}H-^{13}C HSQC) of Xylitol (Xyl)

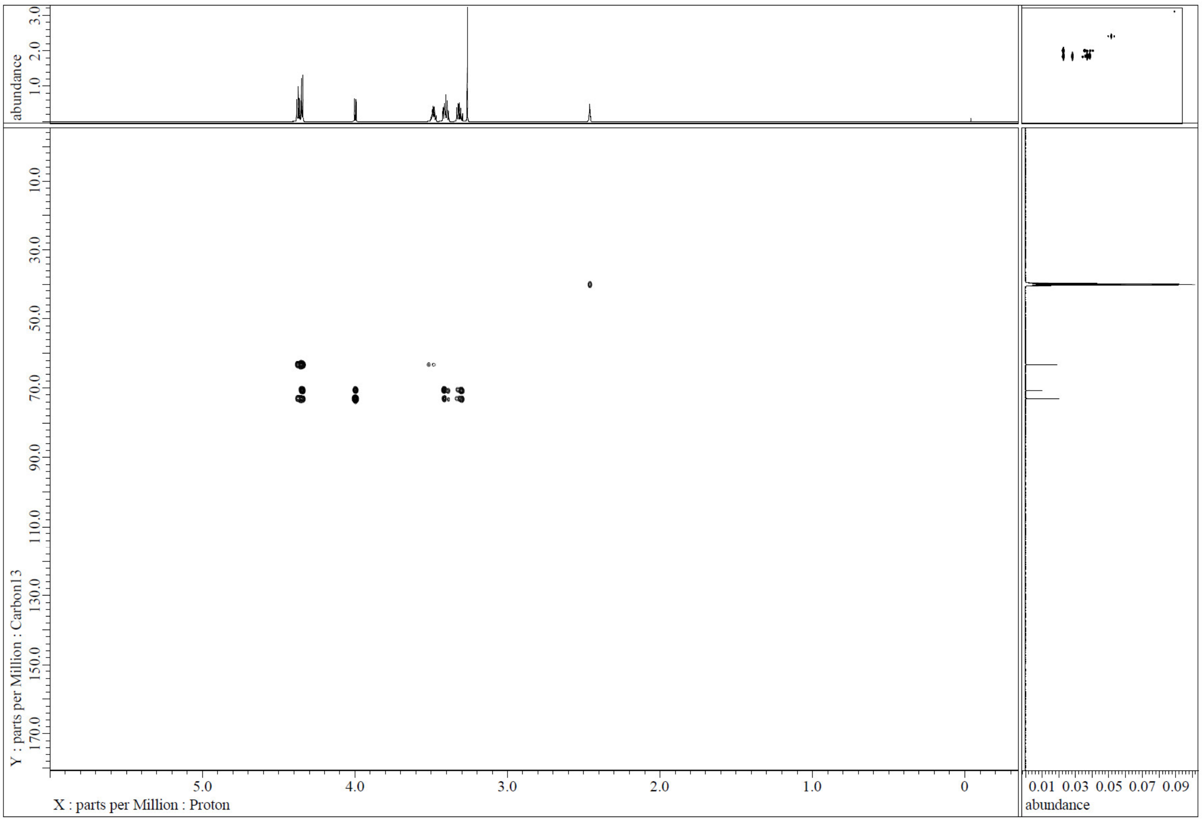

Figure S15. Solution-state 2D NMR (^{1}H-^{13}C HMBC) of Xylitol (Xyl)

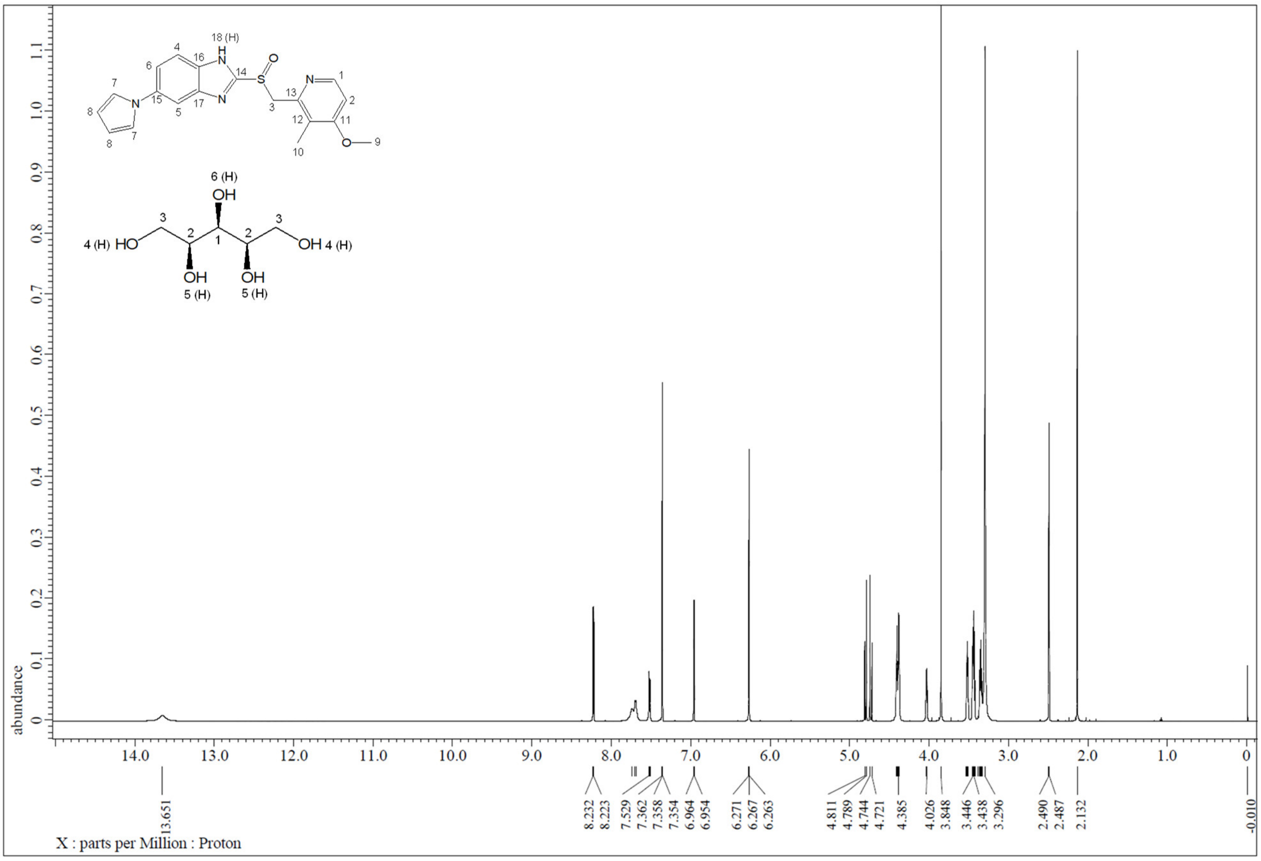

Figure S16. Solution-state ¹H-NMR of Ila/Xyl cocrystal (solvent: DMSO-d⁶)

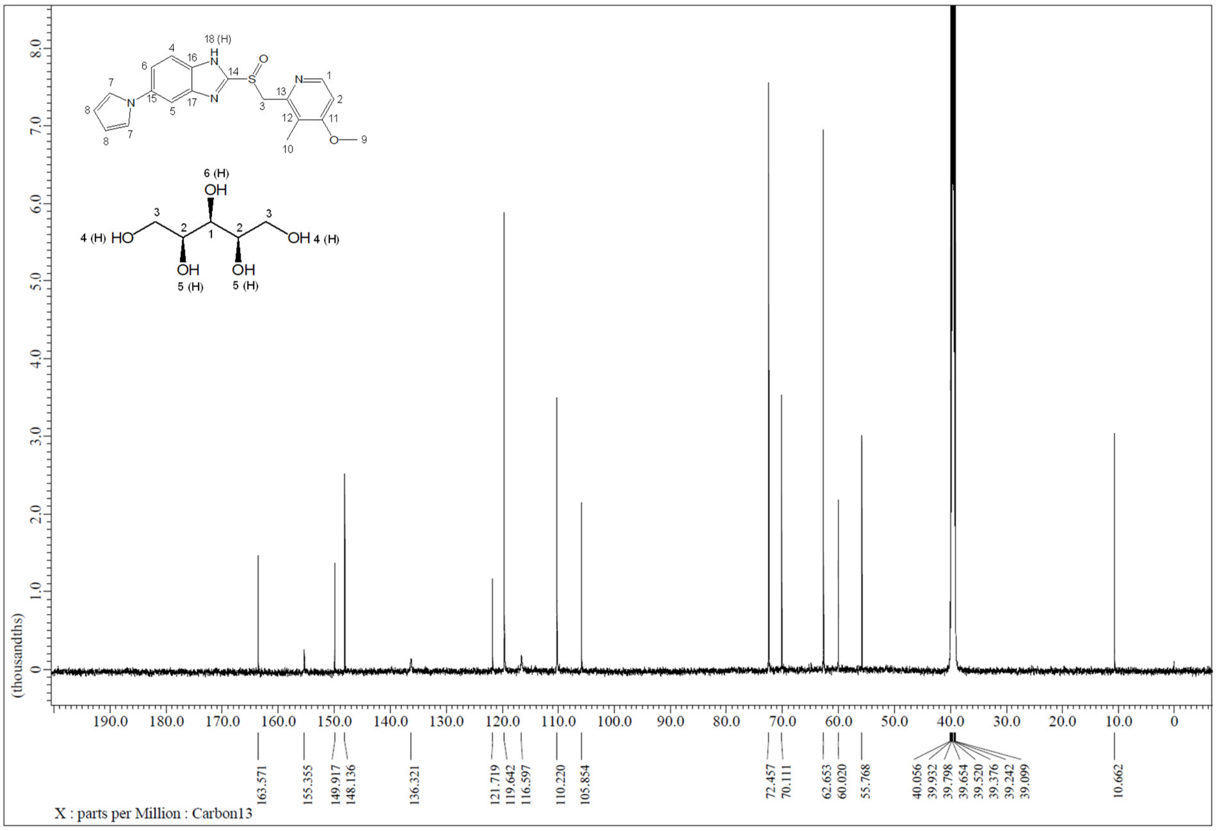

Figure S17. Solution-state ^{13}C-NMR of Ila/Xyl cocrystal (solvent: DMSO-d^6)

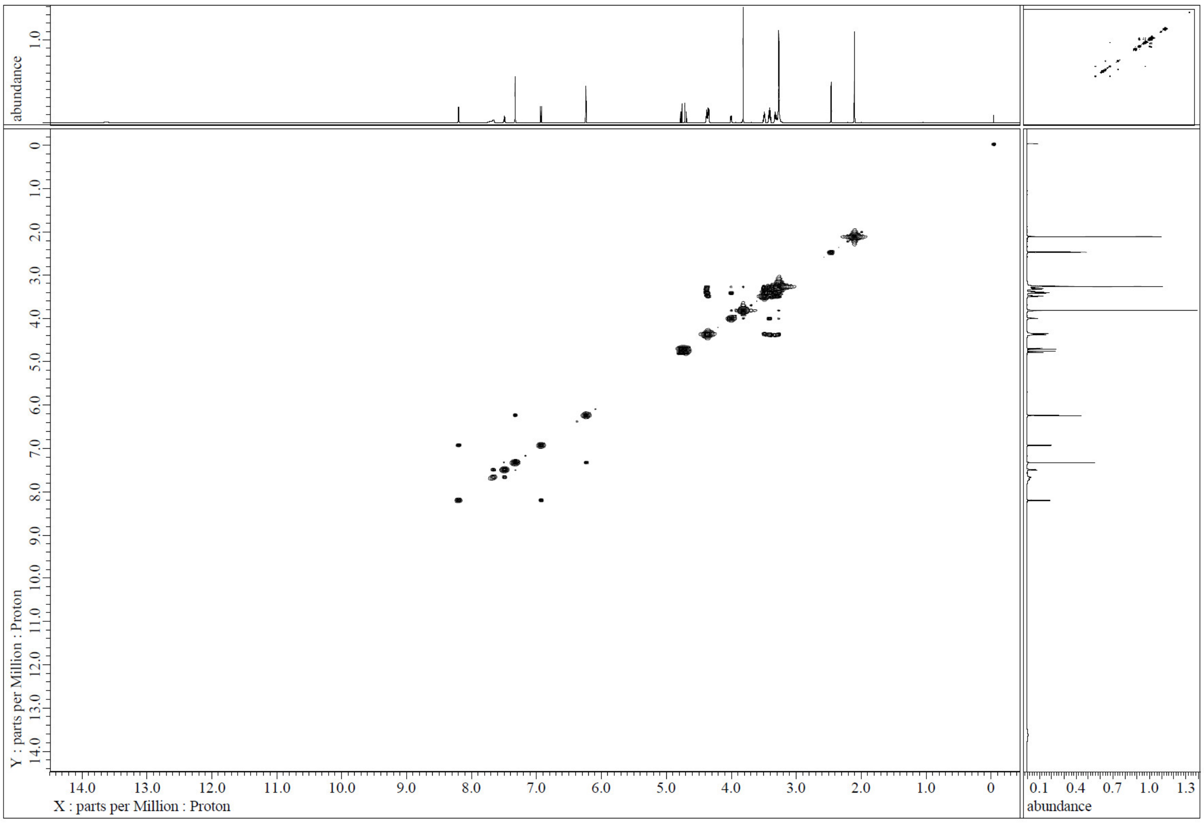

Figure S18. Solution-state 2D NMR (^1H-^1H COSY) of IIa/Xyl cocrystal

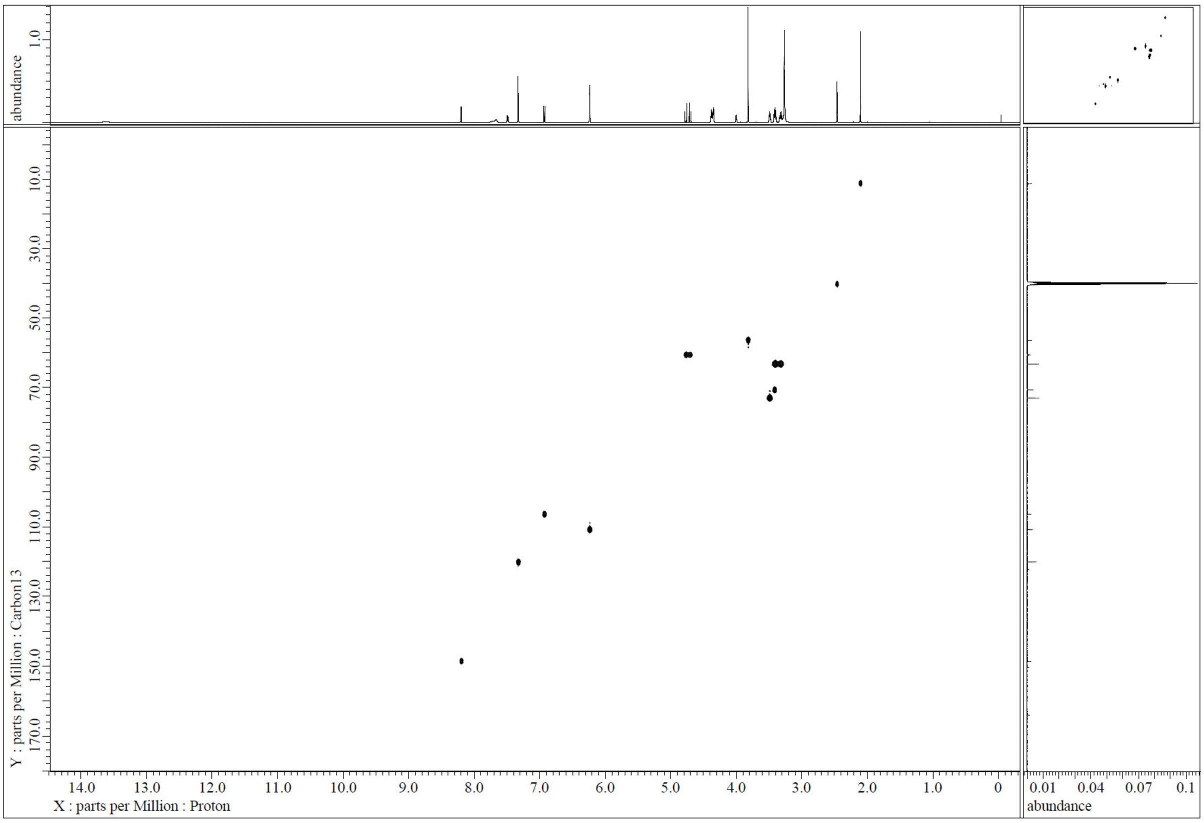

Figure S19. Solution-state 2D NMR (^{1}H-^{13}C HSQC) of Ila/Xyl cocrystal

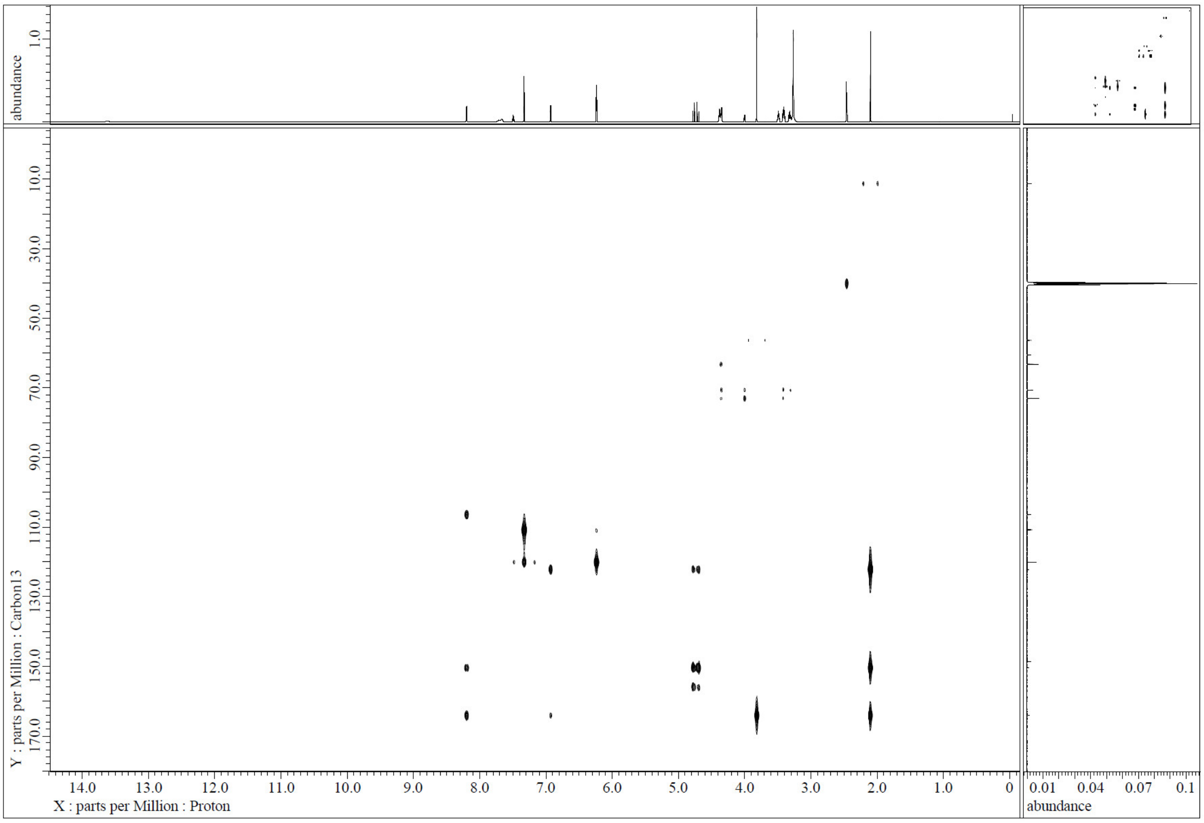

Figure S20. Solution-state 2D NMR (^{1}H-^{13}C HMBC) of Ila/Xyl cocrystal
